# Drug-Encoded Biomarkers for Monitoring Biological Therapies

**DOI:** 10.1371/journal.pone.0137573

**Published:** 2015-09-08

**Authors:** Desislava Tsoneva, Jochen Stritzker, Kristina Bedenk, Qian Zhang, Alexa Frentzen, Joseph Cappello, Utz Fischer, Aladar A. Szalay

**Affiliations:** 1 Department of Biochemistry, Biocenter, University of Würzburg, Würzburg, Germany; 2 Genelux Corporation, San Diego Science Center, San Diego, CA, United States of America; 3 Biocenter, University of Würzburg, Würzburg, Germany; 4 Department of Radiation Oncology, Moores Cancer Center, University of California San Diego, San Diego, CA, United States of America; Swedish Neuroscience Institute, UNITED STATES

## Abstract

Blood tests are necessary, easy-to-perform and low-cost alternatives for monitoring of oncolytic virotherapy and other biological therapies in translational research. Here we assessed three candidate proteins with the potential to be used as biomarkers in biological fluids: two glucuronidases from *E*. *coli* (GusA) and *Staphylococcus sp*. RLH1 (GusPlus), and the luciferase from *Gaussia princeps* (GLuc). The three genes encoding these proteins were inserted individually into vaccinia virus GLV-1h68 genome under the control of an identical promoter. The three resulting recombinant viruses were used to infect tumor cells in cultures and human tumor xenografts in nude mice. In contrast to the actively secreted GLuc, the cytoplasmic glucuronidases GusA and GusPlus were released into the supernatants only as a result of virus-mediated oncolysis. GusPlus resulted in the most sensitive detection of enzyme activity under controlled assay conditions in samples containing as little as 1 pg/ml of GusPlus, followed by GusA (25 pg/ml) and GLuc (≥375 pg/ml). Unexpectedly, even though GusA had a lower specific activity compared to GusPlus, the substrate conversion in the serum of tumor-bearing mice injected with the GusA-encoding virus strains was substantially higher than that of GusPlus. This was attributed to a 3.2 fold and 16.2 fold longer half-life of GusA in the blood stream compared to GusPlus and GLuc respectively, thus a more sensitive monitor of virus replication than the other two enzymes. Due to the good correlation between enzymatic activity of expressed marker gene and virus titer, we conclude that the amount of the biomarker protein in the body fluid semiquantitatively represents the amount of virus in the infected tumors which was confirmed by low light imaging. We found GusA to be the most reliable biomarker for monitoring oncolytic virotherapy among the three tested markers.

## Introduction

The development of the oncolytic virus therapy of cancer and its progress in clinical testing require the development of appropriate and reliable tools, such as reporter genes, to monitor beneficial outcomes as well as potential side effects. Oncolytic virotherapy takes advantage of tumor specific replication of viruses, which ultimately leads to the destruction of cancer cells through direct tumor cell lysis and/or activation of the immune system [1]. Therefore, reporter genes, whose products remain within virus-infected cells can be used for direct imaging of the spatial location and distribution of the (live) drug *in vivo* indicating tumor targeting and a lack of off-target tissue infectivity. However, imaging of patients can be a cost and time consuming endeavor and might not be appropriate for regular monitoring of the oncolytic virotherapy. Therefore, reporter gene products that can be released from the infected cells, either by active secretion or due to tumor cell lysis, would allow easier detection of the biomarker protein in patient samples such as blood and/or other body fluids. Such reporter proteins could be easily assessed repeatedly from the blood and would be a helpful indicator of viral presence in the body. Endogenous biomarkers are limited in such a utility because they may not be specific to the therapeutic, may lack sensitivity, or their baseline levels may fluctuate or vary between individuals. Many of these limitations can be overcome by introducing into the (live) drug a unique reporter gene whose expression is specific to the activity of the therapeutic agent. If the product of the reporter gene is an enzyme, the activity of the enzyme may serve as a biomarker providing a quantifiable predictor of therapeutic performance of the oncolytic treatment.

Recombinant vaccinia virus (rVACV) strains are among the promising candidates with oncolytic properties that are currently being evaluated in clinical trials [1–3]. We previously showed that tissue distribution of rVACV can be assessed in mice by reporter gene-mediated optical- [4,5], optoacoustic- [6], PET- [7,8], and MR-imaging [6]. In addition, we also established a simple, fast and inexpensive blood-based assay to verify rVACV replication in mice [9] and in human patients [10]. The assay is based on the production of *E*. *coli* glucuronidase in infected cells by the engineered oncolytic vaccinia virus strain GLV-1h68. Since the virus is primarily infecting tumor cells, the enzyme is produced upon expression of the *gusA* gene in those cells. Due to virus-mediated cell lysis, it is released and can be detected in the circulation. Using glucuronidase-specific fluorogenic substrates, the enzymatic activity in serum can be quantified and shown to increase over time during the tumor colonization process [9]. The method distinguishes between the *E*. *coli* glucuronidase (GusA) encoded by the virus and the endogenous mammalian glucuronidase present in mice [11] and in the serum of cancer patients. These two enzymes are distinguished on the basis of their different pH optima. The mammalian enzyme has a pH optimum of pH4.8 [12] and has no detectable activity in blood samples when assayed at pH7.4, the pH used to detect GusA activity.

Similarly, the success of certain therapies e.g. mesenchymal stem cell implantation into the heart, depends on the correct spatiotemporal location of the therapeutic agent and therefore would benefit from an imaging monitor that remains permanently with the affected cells or tissues. For other therapies an indirect monitoring by measuring a biomarker, away from the treatment site, is sufficient as an indicator of the biological responses to the therapeutic intervention. For example, release of a biomarker into the blood stream would be appropriate for a gene therapy treatment to correct for example a hematological deficiency, where the therapeutic effect would be widespread throughout the body.

Here we compared the *E*. *coli* beta-glucuronidase (GusA) with other reporter enzymes *in vitro*, in cell culture, as well as in animal experiments. Previously our tests of samples from human cancer patients undergoing oncolytic vaccinia virus therapy in the clinical trial of GL-ONC1 (Clinical grade GLV-1h68) indicated that the amount of glucuronidase detected in serum was near the detection limit of the assay [10]. Therefore, a specific goal of this study was to determine whether the sensitivity of the assay could be improved using either more sensitive substrates and/or enzymes with greater activity. Other markers included in this study were the secreted *Gaussia princeps* luciferase (GLuc) and the cytoplasmic *Staphylococcus sp*. *RLH1* glucuronidase (GusPlus), a glucuronidase with reported higher specific activity than GusA [13–15]. While *Gaussia* luciferase had already been tested in an oncolytic Herpes simplex virus strain [16], to our knowledge, GusPlus has not been used as a biomarker in live animals or patients. In addition, we tested additional fluorogenic and luminogenic substrates for glucuronidases and compared them for improved sensitivity. Our results demonstrated that while the assay for GusPlus activity displayed superior sensitivity *in vitro* and in cell culture, the assay for GusA yielded the best sensitivity *in vivo*.

## Materials and Methods

### Construction of plasmids, GusPlus expressing *E*. *coli* and rVACV

Codon optimized versions of GLuc (GenBank: AAG54095.1) and GusPlus (GenBank: AAK29422.1) were synthesized by GenScript and flanked by *Sal*I and *Pac*I sites. Sequences were cloned into the transfer vectors TK-p11-GLuc and HA-p11-GusPlus respectively. The recombinant viruses GLV-1h351, GLV-1h352 and GLV-1h408 (Table 1) are derivatives of GLV-1h68 [5] and were constructed using transient dominant guanosine phosphoribosyltransferase selection [17].

**Table 1:** The reporter enzymes encoding genes in rVACV strains.

rVACV strain	*F14*.*5L*	*TK*	*HA*
GLV-1h68	**P**_**SEL**_ **-*Ruc-GFP***	P_SEL_-*rTrfR*-P_7.5_-*lacZ*	**P**_**11**_ **-*gusA***
GLV-1h351	non-relevant gene	**P**_**11**_ **-*GLuc***	**P**_**11**_ **-*gusA***
GLV-1h352	non-relevant gene	**(P**_**11**_ **-*GLuc*)**_**inverse**_	**P**_**11**_ **-*gusA***
GLV-1h408	**P**_**SEL**_ ***Ruc-GFP***	P_SEL_ *rTrfR*-P_7.5_ *lacZ*	**P**_**11**_ **-*gusPlus***

The recombinant vaccinia virus strain GLV-1h68 was constructed as specified by Zhang et al. [5] and used as the parental strain for the other virus constructs described in this study. Gene inserts are *Renilla* luciferase-*Aequorea* green fluorescent protein fusion cDNA (*Ruc-GFP*), human transferrin receptor cDNA (*TrfR*), β-galactosidase cDNA (*lacZ*), *E*.*coli* β-glucuronidase cDNA (*gusA*), *Staphylococcus* glucuronidase cDNA (*gusPlus*) and *Gaussia* luciferase cDNA (*GLuc*). Relevant reporter gene constructs used in this study are shown in bold. The human transferrin receptor cDNA is inserted in reverse (rTrfR) and is therefore not expressed in GLV-1h68. Promoters are synthetic early/late promoter (P_SEL_), vaccinia virus early/late P7.5 promoter (P_7.5_) and the vaccinia virus P11 late promoter (P_11_). GLV-1h351 and -1h352 were derived from GLV-1h68 by replacing the P_SEL_-*rTrfR*-P_7.5_-*lacZ* expression cassette at the *TK* locus with the GLuc expression cassette P_11_-*GLuc*, which was inserted in both possible orientations. GLV-1h408 was obtained from GLV-1h68 after replacing the *gusA* expression cassette at the *HA* locus with the P_11_-*gusPlus* expression cassette.

For expression and purification of GusPlus, the open reading frame was PCR-amplified from PCRII-GusPlus using the primers 5'-AAA GGA TCC ATG CTG TAT CCT ATC AAC-3' and 5'-AAA CTG CAG GTC GAC TTA GTT TTT GTA-3'. The resulting fragment was digested with *Bam*HI and *Sal*I and after purification was ligated into the equally cut pET-28a vector. The resulting plasmid pET-28(a)-GusPlus was transformed into *E*. *coli* BL21.

### GusPlus purification

**Fig 1:**
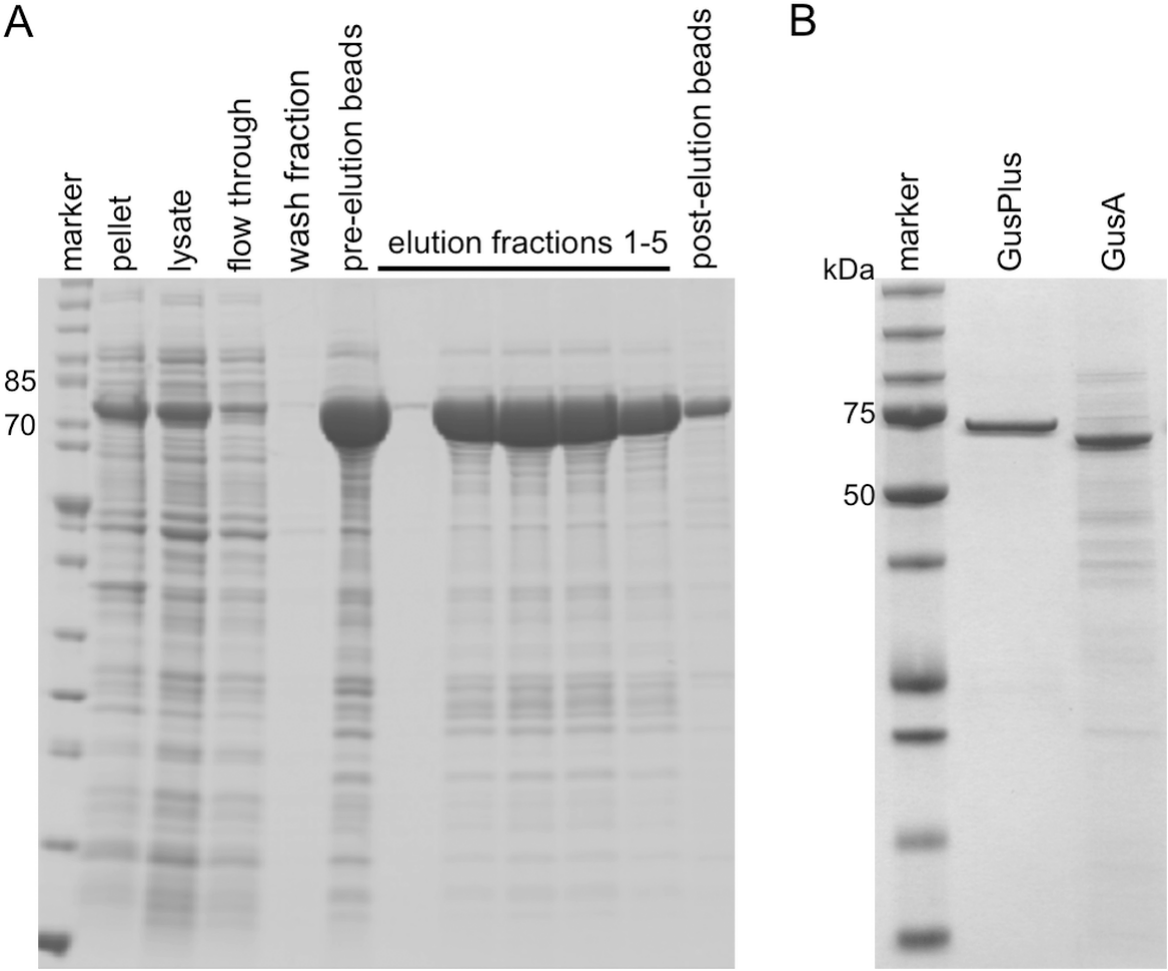
Purification of His-tagged GusPlus. **A)** GusPlus expression was induced in E. coli BL21 pET-28a-GusPlus and the bacteria were harvested by centrifugation (pellet) after overnight culture at room temperature. The bacteria were then lysed (lysate) and loaded on Ni-NTA beads onto which His-tagged GusPlus bound preferentially (GusPlus was clearly reduced in the flow through fraction). The Ni-NTA column was washed (wash fraction) and the beads in the column were shown to have high amounts of bound GusPlus (pre-elution beads). The elution fractions 2–5 contained highly enriched His-tagged GusPlus (post-elution beads). **B)** The combined, dialyzed elution fractions were analyzed by Coomassie-stained SDS-PAGE and determined to be 95.6% His-tagged GusPlus. Commercially available GusA was shown to be 49.3% pure by loading on a Coomassie-stained SDS-PAGE 523 ng of total protein of purified GusPlus (center lane) and 1014 ng of total protein of GusA (right lane). The equal intensities of the GusPlus and GusA bands demonstrated their differences in purity.

*E*. *coli* BL21 pET-28a-GusPlus were grown overnight on a shaker (205 rpm) at 37°C in LB broth. The overnight culture was used to inoculate 500 ml LB broth and was cultured at 37°C and 205 rpm until the optical density at 600 nm reached 0.67. At this time, 500 μl of a 1M Isopropyl β-D-1-thiogalactopyranoside stock solution was used to induce GusPlus production and the culture was incubated shaking (60 rpm) at room temperature overnight. The bacteria were collected by centrifugation and frozen at -80°C. The bacterial pellet was resuspended and sonicated (3 x 3 min) in 15 ml lysis buffer (150 mM NaCl, 50 mM Tris-base, 5 mM β-mercaptoethanol, 5 mM imidazole, proteinase inhibitors, pH 7.5) before cellular debris was removed by centrifugation (25,000 rpm in a 70Ti Beckman Coulter rotor for 40 min at 4°C). The cleared lysate was then added to 500 μl Superflow Ni-NTA beads (Qiagen GmbH, Hilden, Germany) and incubated for 2 h at 4°C with agitation. Beads were then transferred to a column and washed once with 5 ml lysis buffer and 3 times with 5 ml washing buffer (150 mM NaCl, 50 mM Tris-base, 5 mM β-mercaptoethanol, 10 mM imidazole, pH 7.5). The bound protein was eluted using 5x 500 μl elution buffer (150 mM NaCl, 50 mM Tris-base, 5 mM β-mercaptoethanol, 500 mM imidazole, pH 7.5). Elution fractions 2–5 were pooled and dialyzed over night against 150 mM NaCl and 50 mM Tris (pH 7.5). Recovery during purification was evaluated by SDS-PAGE (Fig 1A). The purity of the enzyme was determined to be 95.6% by Coomassie-stained SDS-PAGE analysis using the ImageJ 1.45s software (Fig 1B).

### Cell culture

African green monkey kidney fibroblasts (CV-1, CCL-70™), the human lung carcinoma A549 (CRM-CCL-185™) and the human prostate cancer PC-3 (CRL-1435™) cells were obtained from the American Type Culture Collection (ATCC). CV-1 and PC-3 cells were cultured in Dulbecco’s modified Eagle’s medium (DMEM) (Mediatech, USA) supplemented with 10% fetal bovine serum (FBS) (Mediatech, USA) under standard cell culture conditions (37°C and 5% CO_2_). A549 cells were cultured in RPMI 1640 media (Mediatech, USA) instead of DMEM.

### Viral infection assays

CV-1, A549 and PC-3 cells were seeded onto 24-well plates. After 18–24 hours in culture, the cells were infected with vaccinia virus at a multiplicity of infection (MOI) of 0.005, unless otherwise specified. Infection was performed using medium supplemented with 1% antibiotic-antimycotic solution (10,000 units/ml penicillin G, 10,000 μg/ml streptomycin, 25 μg/ml amphotericin B) (Mediatech, USA) and 2% FBS. Plates were incubated for 1h at 37°C with gentle agitation every 20 min. Afterwards infection medium was replaced by fresh growth medium without phenol red containing 1% antibiotic-antimycotic solution. At 8, 24, 48, 72 and 96 hours post-infection (hpi) the supernatant was removed and stored at -80°C. Fresh medium (1 ml) was added to the infected cells which were then also frozen at -80°C. After thawing, all cell fragments were mechanically removed from the plates by pipetting, followed by ultrasound treatment to completely disrupt cells.

### Animal models

Five to six week old athymic nude mice (Harlan Laboratories, Indianapolis, USA) were each subcutaneously injected onto the right flank with 5x10^6^ A549 lung cancer cells. Animals were monitored daily for any sign of mortality, abnormal mobility, hunched posture or other signs of distress. Tumors were allowed to grow for 16 days before groups of 10 mice were injected into the retro orbital sinus vein with 2x10^6^ pfu/mouse of GLV-1h68, GLV-1h351, or GLV-1h408 respectively. One mouse in the GLV-1h351 group did not show successful tumor colonization (no signal in GLuc- or GusA-assays, absence of plaque forming units in the tumor at the end of the experiment) and was therefore excluded from the statistical analysis of the experiment. *Renilla* luciferase activity in tumors of mice injected with GLV-1h68 or GLV-1h408 was determined weekly by low light imaging. For serum analysis, about 50 μl of blood were collected immediately before and 7, 14, and 21 days after virus injection. Upon euthanization on day 28 after virus injection, more blood was collected.

Half-life time measurements of the proteins were performed in BALB/c mice (Harlan Laboratories, Indianapolis, USA). Groups of 10 (GusA, GusPlus) or 5 (GLuc) mice were injected i.v. with an equivalent of 5 μg purified GusA and GusPlus or 20 μg GLuc per mouse. Blood was collected at different times after injection for marker protein concentration determinations in the serum using the established marker protein assays (see below). Half-life times were calculated using the Excel software plug-in program PK Functions (Joel I. Usansky, Atul Desai, and Diane Tang-Liu, Department of Pharmacokinetics and Drug Metabolism, Allergan, Irvine, USA).

### Ethics statement

These animal studies were approved by and performed in strict accordance with the guidelines of the Institutional Animal Care and Use Committee (IACUC) of Explora Biolabs, San Diego (Approval numbers: EB11-025 and EB13-003).

### Glucuronidase assay

The standard protocol for the glucuronidase assays was performed essentially as described previously [9]. In brief, 5 μl or 10 μl of test or control samples or standard solutions with specified glucuronidase concentration were added to a 384-well plate (black with optical bottom—Thermo Fisher Scientific, Rochester, USA), followed by addition of 75 μl or 70 μl test solution, respectively. The test solution consisted of PBS, 2% FBS supplemented with 0.2 μl 36.5 mM stock solution (in DMSO) of a fluorogenic glucuronidase substrate (see Table 2 for the glucuronidase substrates used in this study). After one hour incubation at 37°C, the fluorescence emission of each well was measured using a Spectra Max M5 (Molecular Devices, Sunnyvale, USA) microplate reader. Results are presented in relative fluorescence units (RFU).

**Table 2:** Glucuronidase assay substrates.

name	abbreviation	excitation wavelength	emission wavelength	source
6-Chloro-4-methylumbelliferyl β-D-glucuronide	Cl-MUGlcU	386 nm	445 nm	Glycosynth, Warrington, UK
4-Methlyumbelliferyl β-D-glucuronide	MUGlcU	365 nm	455 nm	Life Technologies, Carlsbad, USA
Carboxyumbelliferyl β-D-glucuronide	CUGlcU	386 nm	445 nm	Marker Gene Technologies Inc., Eugene, USA
Fluorescein di-β-D-glucuronide	FDGlcU	489 nm	525 nm	Life Technologies, Carlsbad, USA
β-Gus Juice Plus	GusJuice	N/A	no filter	PJK GmbH, Kleinbittersdorf, Germany

In the luminescent assay, the β-Gus Juice Plus (PJK GmbH, Kleinbittersfeld, Germany) substrate was used. In the 384-well plate format, 37.5 μl of β-Gus Juice was added to 5 μl of sample, and the mix was incubated for 1 hour at room temperature before adding 37.5 μl of Triggering reagent. Ten minutes later the luminescence signal was measured for 1000 ms in a Spectra Max M5 microplate reader. Results are presented in relative light units (RLU). Cell culture or serum samples containing high glucuronidase concentrations (readings exceeding the linear range of the assay) were diluted in PBS, 2% FBS prior to testing.

### *Gaussia* luciferase assay

For the standard *Gaussia* luciferase assay we tested 5 μl or 10 μl test samples and standards with the Pierce Gaussia Luciferase Glow Assay Kit (Thermo Scientific, Rockford, USA) or the Gaussia Glow-Juice Kit (PJK GmbH, Kleinbittersfeld, Germany) according to the manufacturer’s instructions, but adjusted to a 384-well format (50 μl or 55 μl total volume). Results are presented in relative light units (RLU).

### *Renilla* luciferase imaging

The activity of *Renilla* luciferase in tumors was analyzed by low light imaging of isoflurane anesthetized mice that were i.v. injected with 10 μg coelenterazine (in 100 μl PBS) 5 minutes before imaging in a small animal low light imager (Bruker, Woodbridge, USA). *Renilla* luciferase signal quantification was carried out using the Molecular Imaging software (Bruker, Woodbridge, USA).

## Statistical analysis and regression curves

Student`s T-test was used to calculate statistical differences between two groups. P-values <0.05 were considered statistically significant. Statistical significance is indicated by asterisks: * (p<0.05); ** (p<0.01); *** (p<0.001). Linear regression curves and R^2^-values were calculated in Microsoft® Excel® 2008 for Mac, Version 12.3.6.

## Results

In this study, three different reporter enzymes (GusA, GusPlus and GLuc) were compared for their use to monitor biological therapies of tumors during oncolytic virotherapy. Key parameters such as specificity, linearity, sensitivity and enzyme stability were analyzed for each purified enzyme. Since *Staphylococcus* glucuronidase GusPlus was not commercially available, we overexpressed the GusPlus protein and purified a His-tagged version in *E*. *coli*. The enzyme purity achieved was 95.6%, which was higher than that of the commercially available *E*. *coli* glucuronidase (purity: 49.3%) (Fig 1).

### *In vitro* evaluation of the specificity, linearity and sensitivity

The activity of *Gaussia* luciferase was assayed using the slightly modified *Gaussia* luciferase Glow Assay kit (Pierce). Glucuronidase activity was assayed using five different substrates, four of which produced a fluorescent signal and one produced a luminescent signal.

The specificity of the assays was determined by running control mouse serum and human plasma samples with substrate but no added reporter enzyme. No non-specific signals above background were observed for any of the assays (data not shown). The background in these assays was determined from the sum of the auto-fluorescence of sample alone and substrate alone. The lack of signal in these tests confirmed that the levels of endogenous glucuronidase in mouse and human serum did not contribute to the signal under the conditions of the assays. Additionally, there was no detectable *Gaussia* luciferase activity in mouse or human serum. Thus, the signals above background obtained in these tests would be specific for the rVACV-expressed reporter enzymes.

**Fig 2:**
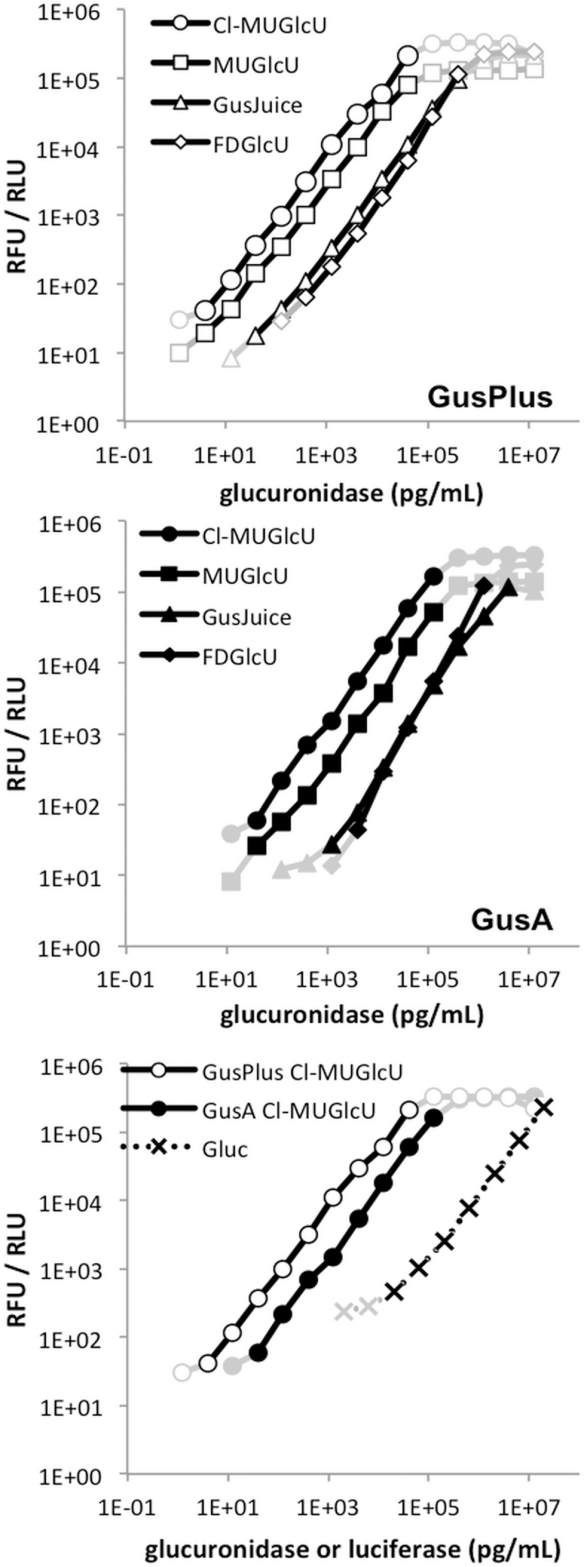
Standard assays of purified marker enzymes. Activity vs enzyme concentration of glucuronidase assays (GusPlus upper chart; GusA middle) with the fluorogenic substrates Cl-MUGlcU, MUGlcU, (CUGlcU was also tested and the results were comparable to MUGlcU, data not shown), and FDGlcU, as well as with the luminogenic GusJuice in human serum are compared. The most sensitive assays for GusPlus and GusA with Cl-MUGlcU and GLuc with the Pierce Gaussia luciferase Glow Assay kit are compared (lower chart). All data points considered were above the limits of detection, as determined for each individual assay. Data points determined to be in the linear range are shown in black while all other data points are shown in grey.

Linearity of the standard assays was evaluated by plotting the relative fluorescence units or the relative light units (for luminogenic assays) over the amount of added enzyme. For these tests, the background fluorescence or luminescence determined for non-enzyme containing samples was subtracted. The linear range was defined as the enzyme concentration range over which the data resulted in a linear regression curve with an R^2^-value >0.99 and the data values used were higher than the limit of quantification (see below). The linear range of all these assays covered at least 3 orders of magnitude (Fig 2). For GLuc, linearity ranged from 20 ng/ml to the highest concentration tested, 20 μg/ml, and the linear range might extend to higher concentrations for this assay.

The sensitivity of the assays was determined by running tests with samples at the low concentration end of the linear range. The limit of detection (LOD) and limit of quantification (LOQ) were defined as 3 and 10 times the standard deviation of the background, respectively. As for sensitivity, the background was determined for each assay as the signal obtained from control samples with no added enzyme. Differences in sensitivity of the assays were observed between enzymes and, for the glucuronidases, between substrates. For GLuc in the standard assay, the LOD was determined to be 2,000 pg/ml. For GusA, the lowest LOD was 25 pg/ml using the Cl-MUGlcU substrate. The LOD for GusPlus was 1 pg/ml in the standard assay using the Cl-MUGlcU substrate. The LOQs for the same assays and substrates were 20,000 pg/ml, 37.5 pg/ml and 12.5 pg/ml for GLuc, GusA and GusPlus, respectively. The LOD for GusPlus could further be reduced to 0.5 pg/ml by extending the assay time from 1 h to 8 h in the standard protocol.

Consistent with these results, GusPlus has been reported to have a higher conversion rate of p-nitrophenyl β-D-glucuronide to p-nitrophenyl than GusA [14,15]. The higher rate of GusPlus catalysis has been attributed to a greater structural flexibility of its protein conformational state due to an increased number of random coils in the secondary structure as well as a greater solvent accessible surface area of the catalytic residues [13]. Our findings are consistent with this hypothesis in that the higher catalytic activity of GusPlus was observed regardless of the substrate being used, 3-fold greater conversion of FDGlcU and 8-fold greater conversion of the GusJuice substrate, compared to GusA.

For both glucuronidases, the sensitivity was highest when assayed with Cl-MUGlcU as substrate. Reduced sensitivity was observed with MUGlcU and CUGlcU followed by GusJuice and FDGlcU. The difference in sensitivity of the assays between the highest and lowest sensitivity substrates was about 2 orders of magnitude. The reason for the lower sensitivity of the assays using the FDGlcU- (and GusJuice-) substrates is probably due to the less effective conversion of FDGlcU compared to Cl-MUGlcU by both glucuronidases, rather than any significant difference in the fluorescence properties of the conversion products.

### Activity of rVACV-expressed biomarker enzymes in cell cultures

A comparison of the different reporter enzymes in cells infected with vaccinia viruses was conducted by placing each gene under the control of the same (P11) promoter. GLV-1h68 contains P11-GusA as its reporter gene, which was inserted into the hemaglutinin gene (HA) locus of the viral genome. The rVACV-strains GLV-1h351 and GLV-1h352 encode the same codon-optimized version of *Gaussia princeps* luciferase (GLuc) under the control of the P11 promoter, inserted into the thymidine kinase gene (TK) locus, however in opposite orientations. GLV-1h408 contains a codon-optimized *gusPlus* gene under the control of the P11 promoter, replacing the *gusA* expression cassette of GLV-1h68 (Table 1). Thus, GLV-1h68 encodes GusA, GLV-1h408 encodes GusPlus, and GLV-1h351 and GLV-1h352 encode both GLuc and GusA, all under control of the same promoter, P11. Therefore, the expression of the reporter enzyme genes in cells infected with these rVACV strains should be proportional to the amount of virus replication in those cells, and the amount of enzyme detected should be directly proportional to the viral titer.

In order to test this, the biomarker enzyme levels of GusA and GLuc were evaluated in cell cultures using A549, PC-3 and CV-1 cell lines, infected with virus at an MOI of 0.005. Culture medium and cells harvested at 8, 24, 48, 72 and 96 hpi were assayed for the activity of the marker enzymes. The data were analyzed individually for each cell type. However, correlations were calculated only from the combined data that were higher than the LOD of each marker protein assay determined in these same experiments.

#### Role of the reporter protein expression cassette orientation in the viral genome

The virus strains GLV-1h351 and GLV-1h352 were used to compare GLuc and GusA activity in the same infected cells in culture. The *GLuc* genes are expressed from the same promoter (P_11_) in each strain, the only difference between them is the orientation of the P_11_-*GLuc* construct in the rVACV genome. Cells infected with each of the two strains showed no statistical difference in the relationship between GLuc activity, GusA activity and virus titer (p>0.44) (S1 Fig). Therefore, the data for the two strains were combined and treated as a single dataset.

#### Reporter enzyme activities and viral titers in cell culture samples

**Fig 3:**
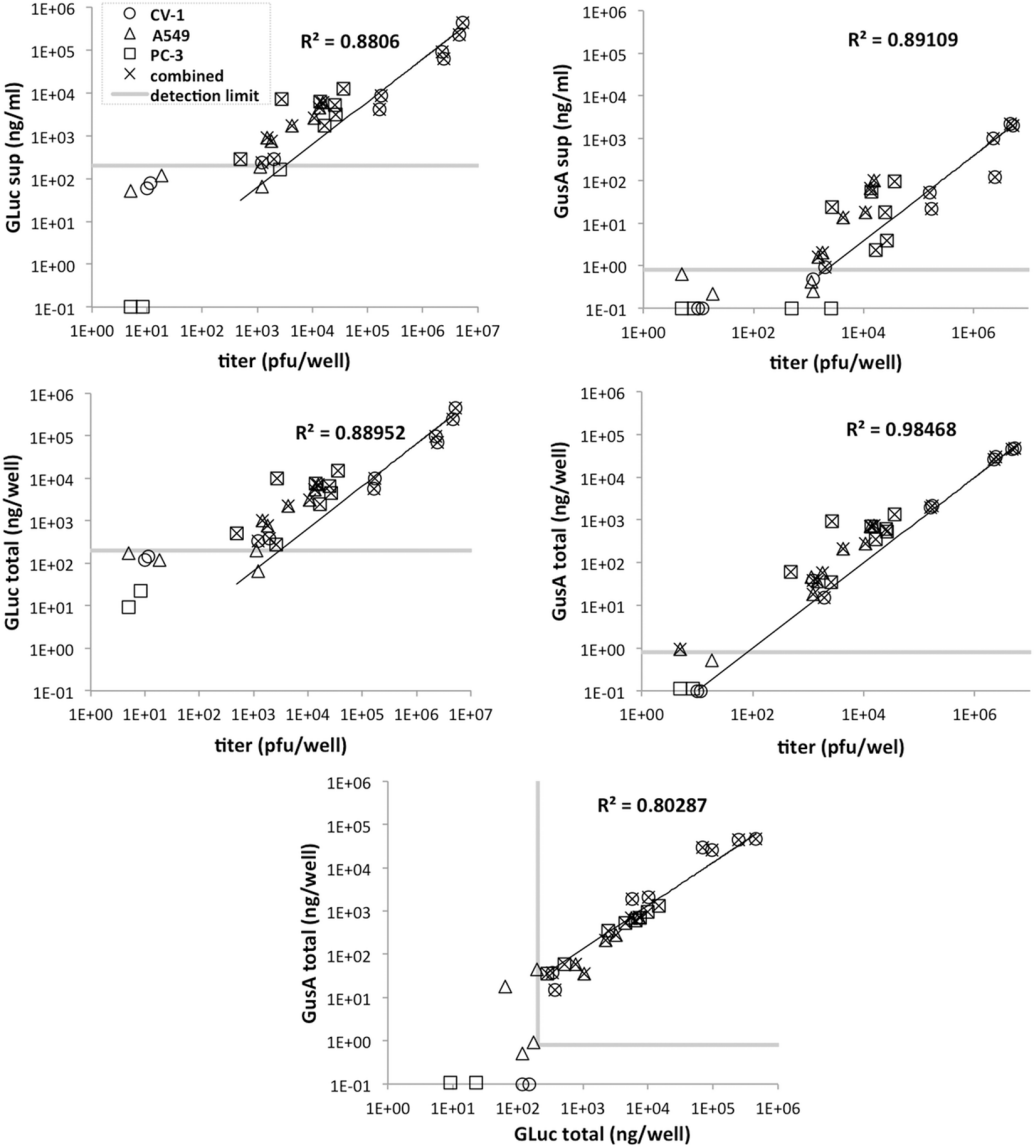
Correlation between GLuc, GusA, and titer in GLV-1h351 and GLV-1h352 infected cells. A549 (triangles), CV-1 (circles) and PC-3 cells (squares) were infected with GLV-1h351 or GLV-1h352 using an MOI of 0.005. The supernatants and cell lysates were assayed for GLuc, GusA and plaque forming units at 8, 24, 48, 72, and 96 hours post infection. The “total” amount per well is the sum of the supernatant and cell lysate values. The grey line(s) in each graph indicates the linear range limit of each assay. Correlation coefficients (R^2^) were calculated using the data points above the linear range limits (indicated by crosses).

Good correlations with correlation coefficients R^2^>0.88 were obtained between the amounts of biomarker enzymes detected in the supernatants or in both the supernatants and cell lysates (as total per well) and the corresponding plaque forming units (Fig 3). The best correlation (R^2^>0.98) occurred with total GusA activity. Therefore, determination of the enzyme biomarker amount of a cell culture sample can be used to estimate the viral titer of the sample. The intersection of the linear regression curve of biomarker enzyme amount and titer and the linear range limit of the biomarker enzyme assay was used to indicate the lower detection limit of titer that can be estimated by assaying the enzyme biomarker amount. For GLuc total amount and GusA supernatant amount, approximately 10^3^ pfu can be detected. For GusA total amount, approximately 10^2^ pfu can be detected.

The relationship between enzyme biomarker activity and viral titer is affected by the nature of the enzyme. Since GusA is a cytoplasmic protein, its release into the supernatants is dependent on cell lysis. Therefore, measurement of GusA activity in the extracellular medium, especially during early infection (8–14 hpi when cells are not yet lysed), does not represent the viral replication correctly. Hence, the correlation between GusA activity in the supernatant and viral titer was only moderate (R^2^ = 0.88). However, the correlation considerably improved when assaying total GusA activity (cells plus supernatant, R^2^ = 0.98). For GLuc, a secreted enzyme, the enzyme activity in the supernatant is not dependent on cell lysis, therefore the correlation with viral titer should not be affected whether the activity is assessed from the supernatant only or from cells and supernatants combined. Indeed, the correlations between viral titer and GLuc supernatant and GLuc total were comparable (R^2^ = 0.88 and R^2^ = 0.89, respectively). Furthermore, because the total activities of the biomarker enzymes each correlated with viral replication, then in turn, they should correlate with each other. Indeed, the correlation coefficient between GLuc and GusA total amount (R^2^ = 0.80) was comparable, and no greater than, the lowest correlation of GLuc and viral titer.

#### GusA and GusPlus as monitoring tools for VACV induced tumor cell death

**Fig 4:**
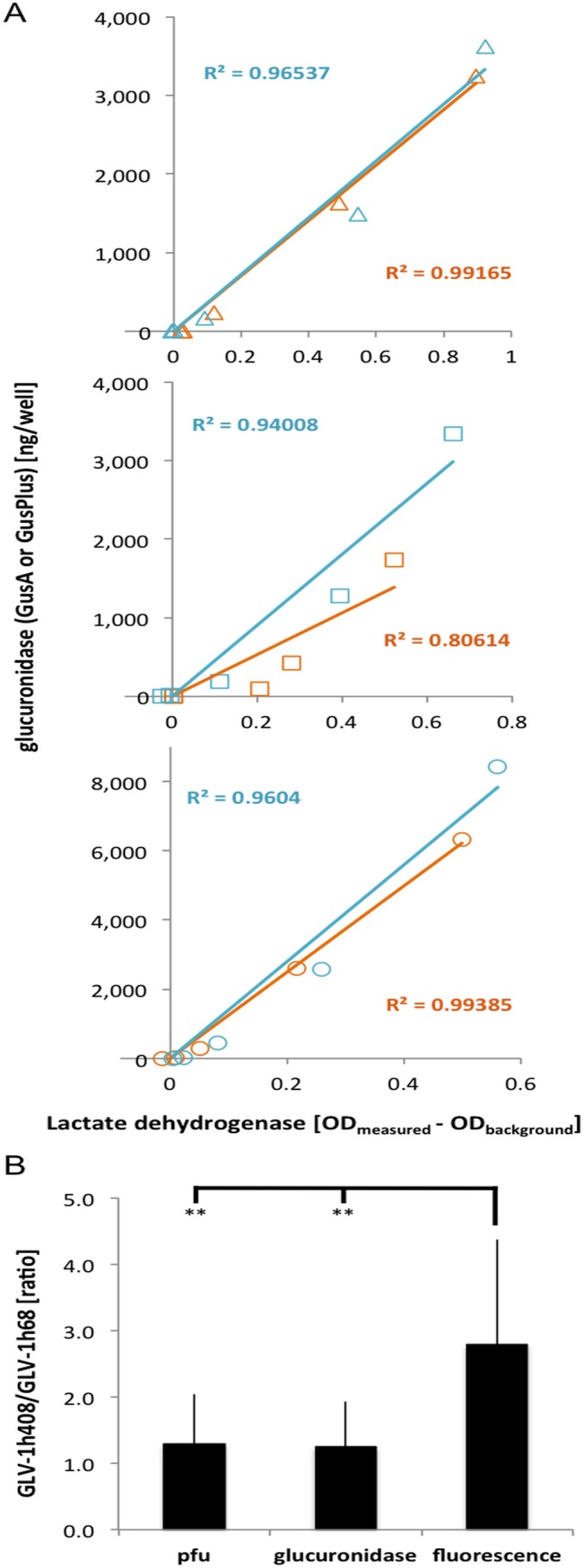
Glucuronidase activity as an indicator of cell death and rVACV titer. **A)** The activities of lactate dehydrogenase and GusA (orange) or GusPlus (blue) in the supernatant of infected A549 (top panel), PC-3 (middle) and CV-1 (lower panel) cells correlated well (R^2^>0.8), which indicates that glucuronidase can be used as an indicator of (virus mediated) cell lysis. **B)** Due to its higher specific activity, GusPlus resulted in higher fluorescent product signal when compared to GusA specific signals in the same cells at the same time post infection. At the same time, the plaque forming units or glucuronidase ratios were similar (ratio about 1) in GLV-1h68 or GLV-1h408 infected cells. Data represent the average plus standard deviation of GLV-1h408/GLV-1h68-ratios obtained from simultaneously infected cells at different times post infection (n = 15). Asterisks indicate statistical differences (p<0.005) between fluorescence ratios and pfu- or glucuronidase ratios.

Since glucuronidases are non-secreted enzymes and will only be released when the infected cells lyse, the glucuronidase signal in the cell culture medium may be a good indicator of virus induced cell lysis. This was investigated by correlating the amounts of GusA or GusPlus activity with lactate dehydrogenase (LDH), an endogenous intracellular enzyme typically used as a marker for cell lysis. Culture supernatants of GLV-1h68 or GLV-1h408 infected cells in culture were assayed for GusA or GusPlus, respectively, and LDH during the course of infection (Fig 4A). There was a good correlation between the GusA and GusPlus amounts and LDH in the supernatants of infected A549, PC-3 and CV-1 cells at all stages of virus infection. The correlation coefficients between GusPlus and LDH ranged from 0.94 for PC-3 cells to 0.96 for A549 and CV-1 cells. For GusA and LDH the correlation coefficients were 0.81 for PC-3, 0.99 for CV-1, and 0.99 for A549 cells. Therefore, the release from cells of the virally expressed biomarker enzymes, GusPlus and GusA, paralleled the release of the cellular enzyme, LDH, as expected since the release was caused by the virus-induced cell lysis.

The results of these experiments again confirmed that GusPlus provided a stronger fluorescent signal per pfu than GusA (Fig 4B). Nonetheless, the amount of biomarker enzyme as determined in the assays of GusPlus and GusA using the purified enzymes as standards yielded comparable enzyme amounts at each time point after simultaneous infection (Fig 4B). This is expected since both enzymes are encoded by genes that are under control of the same P11 promoter, hence the same amount of enzyme synthesis per replicating virus would result.

### Comparison of biomarker enzymes in tumor-bearing animals

**Fig 5:**
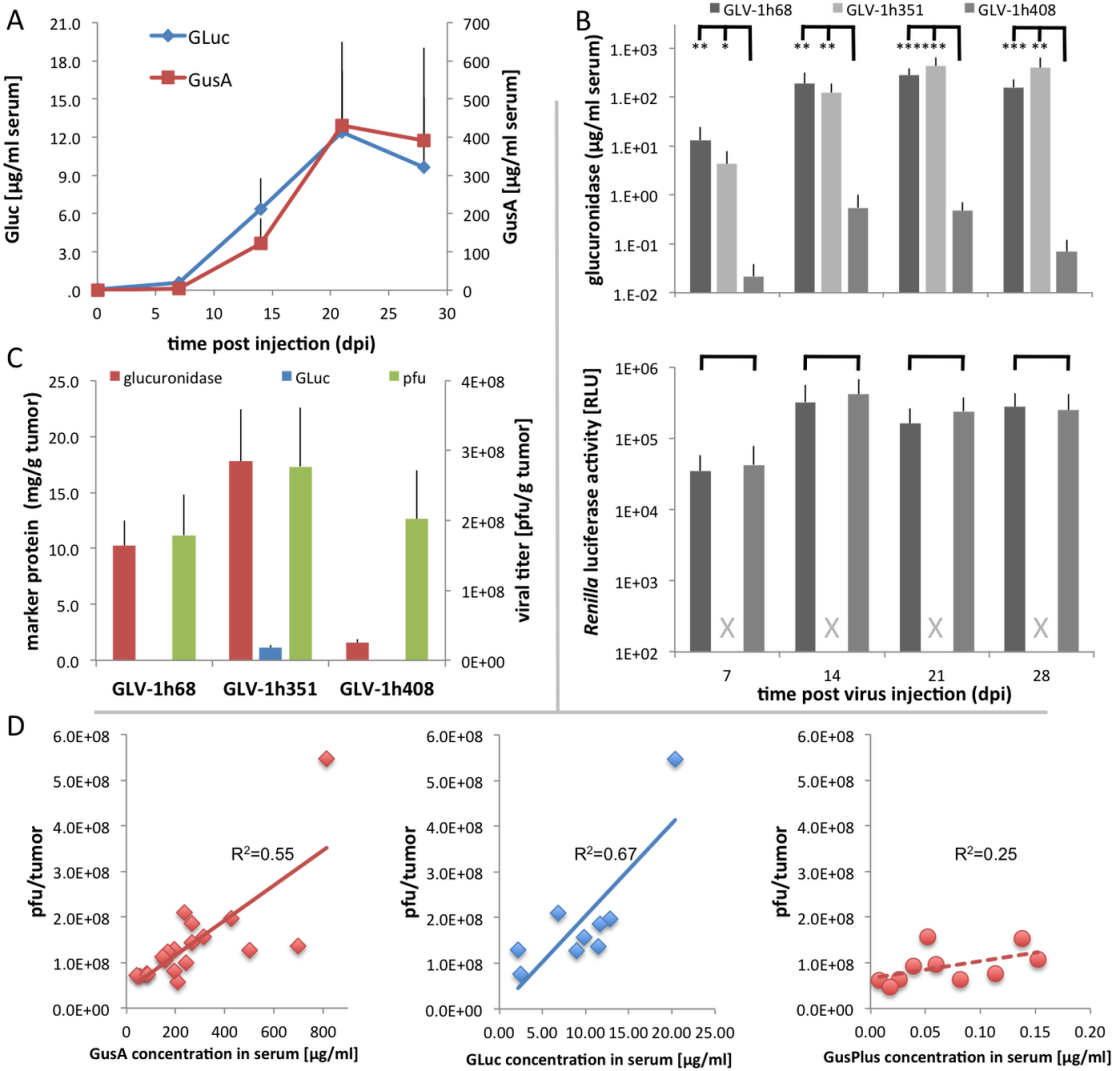
Biomarker enzyme detection in tumor-bearing animals. A549 tumor-bearing animals were injected with GLV-1h68, GLV-1h351 or GLV-1h408. Every week, serum was isolated and tested for biomarker enzyme activity. **A)** GLV-1h351 injected mice were analyzed for the presence of GLuc and GusA activity. **B)** Significantly less GusPlus activity in serum of GLV-1h408 injected mice was observed at all time points compared to GusA in GLV-1h68 and GLV-1h351 injected mice (upper chart). At the same time points, no differences were observed in *Renilla* luciferase activity in GLV-1h68 and GLV-1h408 injected mice (lower chart). Grey crosses indicate that GLV-1h351 injected mice were not imaged for *Renilla* luciferase activity. **C)** Analysis of biomarker enzymes and viral titers in tumors at the end of the experiment. The activities of GLuc and GusPlus are clearly underrepresented relative to viral titers compared to GusA. **D)** Individual mouse data from samples that were obtained at the end of the experiment (28 dpi).

A main objective of this work was to evaluate the use of the drug-encoded marker proteins for monitoring of oncolytic virotherapy in patients. We therefore injected tumor-bearing nude mice with GLV-1h68, GLV-1h351 or GLV-1h408 and assayed for the presence of the biomarker enzyme in serum over a period of 4 weeks. Unexpectedly in mice injected with GLV-1h351, the concentration of GusA in serum was significantly higher, on average 7-fold (7 dpi) to 40-fold (28 dpi), than the GLuc-concentration (Fig 5A). Interestingly, in mice injected with GLV-1h351, levels of GLuc in serum increased more rapidly than the GusA levels (Fig 5A), which was most evident at 14 dpi. This occurred consistently in 8 of the 9 mice examined (S2 Fig). In contrast to the secreted GLuc protein, GusA is a cytoplasmic protein released into the serum only upon tumor cell lysis, and a delay in the levels of GusA in serum relative to GLuc can be explained by this fact. At 21 dpi, the levels of both enzymes reached their maxima and similarly declined at 28 dpi.

A comparison of the amount and timing of increase of glucuronidase serum levels in A549 tumor-bearing mice was examined for each of the three viruses. Glucuronidase levels in serum showed comparable increases at 7, 14 and 21 dpi and no statistically significant differences between animals injected with GLV-1h68 or GLV-1h351 (both expressing GusA) (Fig 5B, upper panel). Surprisingly, however, the glucuronidase serum levels of mice injected with GLV-1h408 (expressing GusPlus) were significantly less and increased only through 14 dpi, declining thereafter to 28 dpi. This was unexpected since in cell culture GusPlus levels were greater than GusA in the cell lines tested, including A549 cells. We examined whether *in vivo* GLV-1h408 might be less effective in colonizing tumors than GLV-1h68. Low light imaging of the GLV-1h68 and GLV-1h408 injected mice was conducted to measure the amount of light emission due to the production of *Renilla* luciferase, which was expressed by both viruses. No substantial differences between GLV-1h68 or GLV-1h408 colonized tumors were observed by *Renilla* luciferase imaging over the whole experiment (Fig 5B, lower panel). This indicated that the concentration of virus within the tumors was comparable in both groups.

Biomarker enzyme and vaccinia virus titer were also analyzed in the tumors at the end of the experiment (Fig 5C). Consistent with the results from the serum and the *Renilla* luciferase imaging, the glucuronidase activity in GLV-1h68 colonized tumors (GusA) was much higher than the activity in tumors colonized with GLV-1h408 (GusPlus), while the virus titers were comparable. Moreover, we analyzed data of samples from individual mice obtained 28 dpi (Fig 5D). The results show correlation between the number of plaques/tumor and GusA (R^2^ = 0.55) and GLuc (R^2^ = 0.67), respectively. The correlation between GusPlus in the serum and the number of plaques in tumors for GLV-1h408 was less pronounced (R^2^ = 0.25).

In both analyses of serum and colonized tumors, the activity of GusA was considerably greater than GLuc and GusPlus, although all enzymes were expressed under the same promoter. An explanation could be a difference in the stability of the biomarker enzymes *in vivo*.

#### Determination of reporter enzymes half-life in mice

Consequently, we analyzed the serum half lives of GusA, GLuc and GusPlus in BALB/c mice. Purified enzymes were injected intravenously, serum was collected at various time points post-injection, assayed for glucuronidase or GLuc activity, and the circulation half-life times were determined. Compared to GusA (half-life: 69.6 ± 8.6 min) GLuc and GusPlus had about one sixteenth (4.3 ± 0.4 min) and one third (21.7 ± 6.5 min) half-life times respectively (Table 3).

**Table 3:** Properties of the different marker proteins and their assays.

	GLuc	GusA	GusPlus
LOD^*^	30–100 pg	2 pg	0.08 pg
linear range	> 3 logs^**^	3.5 logs	3.5 logs
localization	secreted	cytoplasmic	cytoplasmic
*In vivo* Half-life time (min)	4.3 ± 0.4 (n = 5)	69.6 ± 8.6 (n = 10)	21.7 ± 6.5 (n = 12)

* Limit of detection for the described standard assays. For the glucuronidases, the detection limit was lower when incubation time was increased.

** The maximum concentration tested here was 1 μg/assay. According to Tannous, the linear range was at least 5 orders of magnitude [18].

Notably, the observed 4.3 min half-life time for GLuc was shorter than the 20 min, which were reported previously [19]. The reason for this difference may be that we used purified GLuc protein instead of cell culture supernatant, as was used in the published study, and our analysis was done in BALB/c mice instead of nude mice. Nevertheless, the considerably greater half-life time of GusA in serum compared to GLuc and GusPlus allowed a greater accumulation of GusA at equivalent states of virus replication. This translated into a more sensitive monitor of virus replication (and tumor cell lysis) than assays of GLuc and GusPlus.

#### Glucuronidase assay (GusA) in human blood and plasma

Finally, we evaluated the performance of the glucuronidase assay when GusA is spiked into fresh human blood and blood plasma, respectively. Non-specific binding of glucuronidase to blood cells or inactivation of GusA-activity in plasma, for example, would make the assay useless for identification of GusA in human patients. Therefore, we obtained EDTA-blood from 4 healthy donors (San Diego Blood Bank, San Diego, CA, USA). The blood was divided in 2 aliquots, one of which was used to obtain blood plasma. Both EDTA-blood and plasma were spiked with varying concentrations of GusA. The EDTA-blood samples were then centrifuged and the supernatant was used in the glucuronidase assay, which was performed as described using Cl-MUGlcU as substrate with the difference that the assay was performed in a 96-well plate format (instead of 384-well plates) in a total volume of 150 μl that included 10 μl sample volume. Each sample and GusA-concentration was analyzed in triplicate. In parallel, we analyzed GusA-spiked PBS with 2% FBS (Assay Matrix) samples.

**Fig 6:**
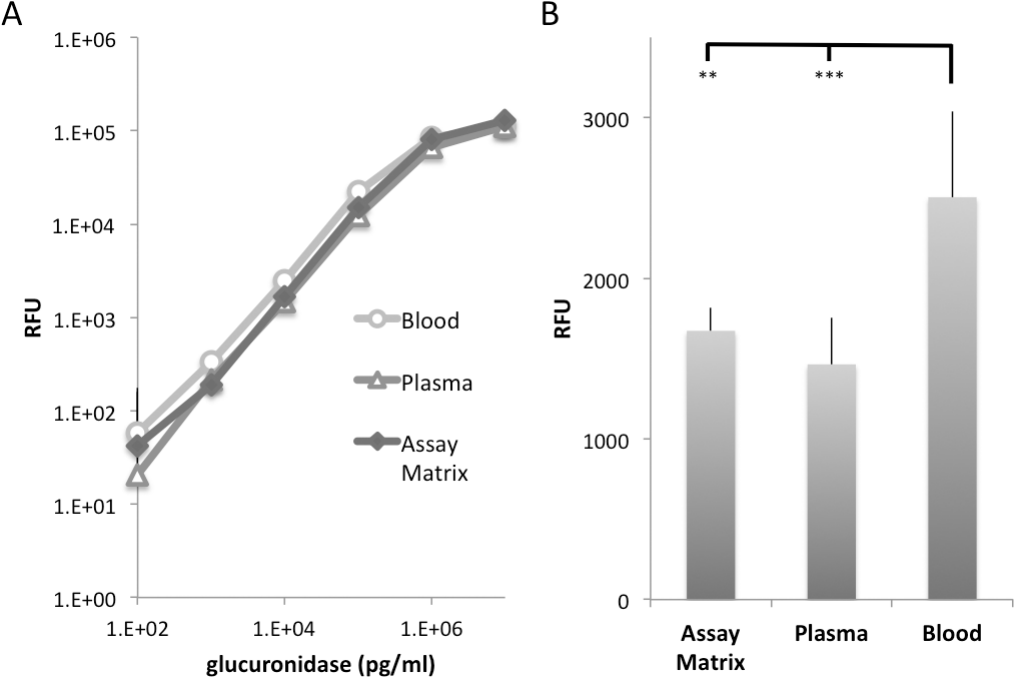
Glucuronidase assay in human blood and plasma. (A) GusA was spiked at varying concentrations into assay matrix (PBS with 2% FBS), human plasma and human blood respectively. A glucuronidase assay of the samples was performed using Cl-MUGlcU as substrate. The supernatant of spiked blood was used in the glucuroronidase assay after centrifugation (7 min, 3000 g). Mean plus standard deviation of 4 samples is shown with each concentration of glucuronidase tested in triplicate (n = 12 for each data point). (B) The data for 10^4^ pg/ml glucuronidase are shown in detail. The fluorescence signal derived from the blood sample is significantly higher compared to the signal that was obtained after spiking the same amount of GusA into identical volumes of assay matrix and plasma respectively. No significant difference was observed between the samples of the latter two groups.

The results in Fig 6A clearly show that neither blood nor serum negatively affected the fluorescence signal that was obtained from the assay. However, the fluorescence signal from the blood samples was significantly higher than the plasma and assay matrix samples (Fig 6B). This can be explained by the fact that normal hematocrit levels in humans are about 36 to 50%. As glucuronidase does not enter the blood cells, the concentration in the supernatant is about 1.6 to 2.0 fold higher when the enzyme is spike into a given volume of blood, compared to spiking into assay matrix or plasma. In conclusion, we showed that the GusA assay is not adversely affected by blood components including human blood cells or human blood plasma.

## Discussion

For the intended application to use rVACV-encoded enzymes as a monitor of oncolytic tumor therapy by measuring the serum activity, the GusA was better suited than GusPlus and GLuc. The greater circulation half-life time *in vivo* of GusA allowed increased accumulation of the enzyme concentration in serum. GusA also had the additional benefit over GLuc of its dependency on cell lysis for release into the circulation. Thus, GusA activity in serum was an indicator not only of rVACV infection and replication, but also tumor cell lysis. In patients the activity of the biomarker in the blood would be a more direct indicator of virus-mediated oncolysis than LDH-measurements. As a monitor of oncolytic therapy, information pertinent to all the characteristics mentioned above would be extremely valuable. In this regard, it was also very important to show that human blood does not have a negative impact on the performance of the GusA-assay.

We did not quantify the protein amounts of GLuc and GusA in GLV-1h351 and GLV-1h352 infected cells, therefore we cannot exclude that the expression levels of GusA and GLuc may be different. Even though the *GLuc* and *gusA* genes were under control of the same promoter (P_11_), the reporter genes are located at different loci in the genome of GLV-1h351 and GLV-1h352. Previously it was reported that the position of a Firefly luciferase gene insertion resulted in expression level differences in Western Reserve strain based constructs that expressed the HSV thymidine kinase but were deficient for the vaccinia virus thymidine kinase [20]. We however, have not observed differences in gene expression between virus strains that encoded fluorescent proteins under control of identical (non-P_11_) promoters at different gene loci within the viral genome of Lister-based vaccinia virus strains (data not shown).

Despite its lesser sensitivity, GLuc might still be a useful biomarker for virotherapy of tumors. Since GLuc is actively secreted from infected cells in the absence of tumor cell lysis, a comparison of GLuc levels with GusA levels might in combination provide information that would not be possible with each marker alone. For example, therapeutic efficacy of viral treatments in which tumor cell infection and virus replication did not lead directly to oncolysis, but rather to anti-tumor immunostimulation, a monitor of tumor cell infection in the absence of cell lysis would be beneficial. This will be investigated in future studies.

Other markers such as carcinoembryonic antigen (CEA) have also been used to monitor oncolytic viruses [21]. In our experience, CEA is not an ideal biomarker for biological therapies for the following reasons. CEA is a human protein that is detectable at low levels even in healthy individuals. In certain cancer patients (e.g. colorectal cancer), CEA levels are upregulated and it has also been described that heavy smokers have upregulated CEA levels. This makes it very problematic to correlate elevated CEA levels in biological therapies with actual therapeutic effects. In addition, the CEA cDNA has a domain, which is repeated 3 times with very similar / partly identical sequence stretches. When we produced a CEA-encoding recombinant vaccinia virus, we frequently observed recombination between those sequence stretches, which resulted in a heterogenous population of viruses with varying numbers (1–3) of the sequence domain (Nube et al., data not shown).

For *in vitro* use and in cell cultures, GusPlus with its greater specific enzyme activity, lesser limit of detection in conjunction with the more fluorogenic substrates of the coumarin family (Cl-MUGlcU, MUGlcU and CUGlcU), and high range of linearity was the superior marker. The excellent correlation of glucuronidase activity with viral titer (which we show here for the first time) supports the replacement of the labor-intensive and time-consuming plaque assay that is commonly used to monitor viral replication by a fluorogenic enzymatic assay. The fact that we tested 5 glucuronidase substrates from 3 different compound classes and that the limit of detection for those compounds varied over 2 orders of magnitude while the linear range for all of the substrates was >3 orders of magnitude, makes the described assays a very versatile tool for quantification of replication competent biological agents, including oncolytic viruses. The extreme sensitivity of the GusPlus assay, which we analyzed here, might also be used for the development of e.g. an ultra-sensitive ELISA employing GusPlus as a reporter instead of horseradish peroxidase (HRP) as a conjugate to secondary antibodies for applications in protein detection methods (Western blot, ELISA), especially for proteins of low abundance. Considering the HRP detection limit of 1.7 pg/ml (http://www.piercenet.com/guide/guide-elisa-substrates) and a molecular weight of 44kDa, GusPlus with an LOD of 0.5 pg/ml (40 fg/80μl assay) and a molecular weight of 69.5kDa for the His-tagged GusPlus, the glucuronidase can be detected about 5-times more sensitively on a per molecule basis.

All three enzymes are promising candidates to fulfill rapidly growing demand for sensitive and reliable monitoring tools in the translational research and the clinical testing not only in the field of the oncolytic virotherapy but also of other biological therapies such as gene and stem cell therapy. The choice of the monitoring method is strongly dependent on the type and the strategy of the biological therapy. Secreted biomarkers (such as GLuc) are useful for monitoring of cell survival as only actively secreting cells could be detected. The release of cytoplasmic enzymes such as glucuronidases indicates cell lysis, which can either indicate failure of therapy (e.g. stem cell therapy) or successful therapy as in our case.

## Supporting Information

S1 Fig The orientation of the GLuc expression cassette inserted into the TK-locus of rVACV had no effect on the expression levels of the reporter enzyme or on the viral replication efficacy. Cells (A549, CV-1 and PC-3) infected with either GLV-1h351 (blue squares) or GLV-1h352 (orange circles) produced comparable relationships of GLuc activity, GusA activity or virus titer between cells infected with GLV-1h351 or GLV-1h352. No significant differences were observed (p>0.47).
(TIF)

S2 Fig GLuc and GusA activities in serum of individual mice over time. In 8 out of 9 mice, the GLuc concentration as determined by its activity increased faster than the GusA concentration (see data at 14 days post injection).
(TIF)

## References

[1] Chen NG, Szalay AA (2010) Oncolytic vaccinia virus: a theranostic agent for cancer. Future Virology 5: 763–784.

[2] Kirn DH, Thorne SH (2009) Targeted and armed oncolytic poxviruses: a novel multi-mechanistic therapeutic class for cancer. Nat Rev Cancer 9: 64–71. 10.1038/nrc2545 19104515

[3] Heo J, Reid T, Ruo L, Breitbach CJ, Rose S, Bloomston M, et al (2013) Randomized dose-finding clinical trial of oncolytic immunotherapeutic vaccinia JX-594 in liver cancer. Nat Med 19: 329–336. 10.1038/nm.3089 23396206 PMC4268543

[4] Yu YA, Shabahang S, Timiryasova TM, Zhang Q, Beltz R, Gentschev I, et al (2004) Visualization of tumors and metastases in live animals with bacteria and vaccinia virus encoding light-emitting proteins. Nat Biotechnol 22: 313–320. 14990953 10.1038/nbt937

[5] Zhang Q, Yu YA, Wang E, Chen N, Danner RL, Munson PJ, et al (2007) Eradication of solid human breast tumors in nude mice with an intravenously injected light-emitting oncolytic vaccinia virus. Cancer Res 67: 10038–10046. 17942938 10.1158/0008-5472.CAN-07-0146

[6] Stritzker J, Kirscher L, Scadeng M, Deliolanis NC, Morscher S, Symvoulidis P, et al (2013) Vaccinia virus-mediated melanin production allows MR and optoacoustic deep tissue imaging and laser-induced thermotherapy of cancer. Proc Natl Acad Sci U S A 110: 3316–3320. 10.1073/pnas.1216916110 23401518 PMC3587225

[7] Chen N, Zhang Q, Yu YA, Stritzker J, Brader P, Schirbel A, et al (2009) A novel recombinant vaccinia virus expressing the human norepinephrine transporter retains oncolytic potential and facilitates deep-tissue imaging. Mol Med 15: 144–151. 10.2119/molmed.2009.00014 19287510 PMC2654849

[8] Haddad D, Chen NG, Zhang Q, Chen CH, Yu YA, Gonzalez L, et al (2011) Insertion of the human sodium iodide symporter to facilitate deep tissue imaging does not alter oncolytic or replication capability of a novel vaccinia virus. J Transl Med 9: 36 10.1186/1479-5876-9-36 21453532 PMC3080806

[9] Hess M, Stritzker J, Hartl B, Sturm JB, Gentschev I, Szalay AA (2011) Bacterial glucuronidase as general marker for oncolytic virotherapy or other biological therapies. J Transl Med 9: 172 10.1186/1479-5876-9-172 21989091 PMC3207905

[10] Mansfield D, Yu YA, Chen NG, Stritzker J, Szalay AA, Harrington KJ (2013) Validation of Biomarkers of Intravenously Administered Oncolytic Vaccinia Virus in a Phase I Trial. Replicating Oncolytic Virus Therapeutics 2013. Quebec, Canada.

[11] Ruiz-Hernandez E, Hess M, Melen GJ, Theek B, Talelli M, Shi Y, et al (2014) PEG-pHPMAm-based polymeric micelles loaded with doxorubicin-prodrugs in combination antitumor therapy with oncolytic vaccinia viruses. Polymer Chemistry. 10.1039/C3PY01097J PMC3836408 24518685

[12] Zenser TV, Lakshmi VM, Davis BB (1999) Human and Escherichia coli beta-glucuronidase hydrolysis of glucuronide conjugates of benzidine and 4-aminobiphenyl, and their hydroxy metabolites. Drug Metab Dispos 27: 1064–1067. 10460807

[13] Arul L, Benita G, Balasubramanian P (2008) Functional insight for beta-glucuronidase in Escherichia coli and Staphylococcus sp. RLH1. Bioinformation 2: 339–343. 18685721 10.6026/97320630002339 PMC2478733

[14] Broothaerts W, Mitchell HJ, Weir B, Kaines S, Smith LM, Yang W, et al (2005) Gene transfer to plants by diverse species of bacteria. Nature 433: 629–633. 15703747 10.1038/nature03309

[15] Jefferson RA, Harcourt RL, Kilian A, Wilson KJ, Keese PK (2002) Microbial β-glucuronidase genes, gene products and uses thereof.

[16] Browne AW, Leddon JL, Currier MA, Williams JP, Frischer JS, Collins MH, et al (2011) Cancer screening by systemic administration of a gene delivery vector encoding tumor-selective secretable biomarker expression. PLoS One 6: e19530 10.1371/journal.pone.0019530 21589655 PMC3092745

[17] Falkner FG, Moss B (1990) Transient dominant selection of recombinant vaccinia viruses. J Virol 64: 3108–3111. 2159565 10.1128/jvi.64.6.3108-3111.1990 PMC249504

[18] Tannous BA (2009) Gaussia luciferase reporter assay for monitoring biological processes in culture and in vivo. Nat Protoc 4: 582–591. 10.1038/nprot.2009.28 19373229 PMC2692611

[19] Wurdinger T, Badr C, Pike L, de Kleine R, Weissleder R, Breakefield XO, et al (2008) A secreted luciferase for ex vivo monitoring of in vivo processes. Nat Methods 5: 171–173. 10.1038/nmeth.1177 18204457 PMC2699561

[20] Coupar BE, Oke PG, Andrew ME (2000) Insertion sites for recombinant vaccinia virus construction: effects on expression of a foreign protein. J Gen Virol 81: 431–439. 10644842 10.1099/0022-1317-81-2-431

[21] Phuong LK, Allen C, Peng KW, Giannini C, Greiner S, TenEyck CJ, et al (2003) Use of a vaccine strain of measles virus genetically engineered to produce carcinoembryonic antigen as a novel therapeutic agent against glioblastoma multiforme. Cancer Res 63: 2462–2469. 12750267

