# Supplementary figures and images for: Drug-Encoded Biomarkers for Monitoring Biological Therapies
Source: PLoS One. 2015 Sep 8;10(9):e0137573. doi: 10.1371/journal.pone.0137573 (PMC4562523; doi:10.1371/journal.pone.0137573)

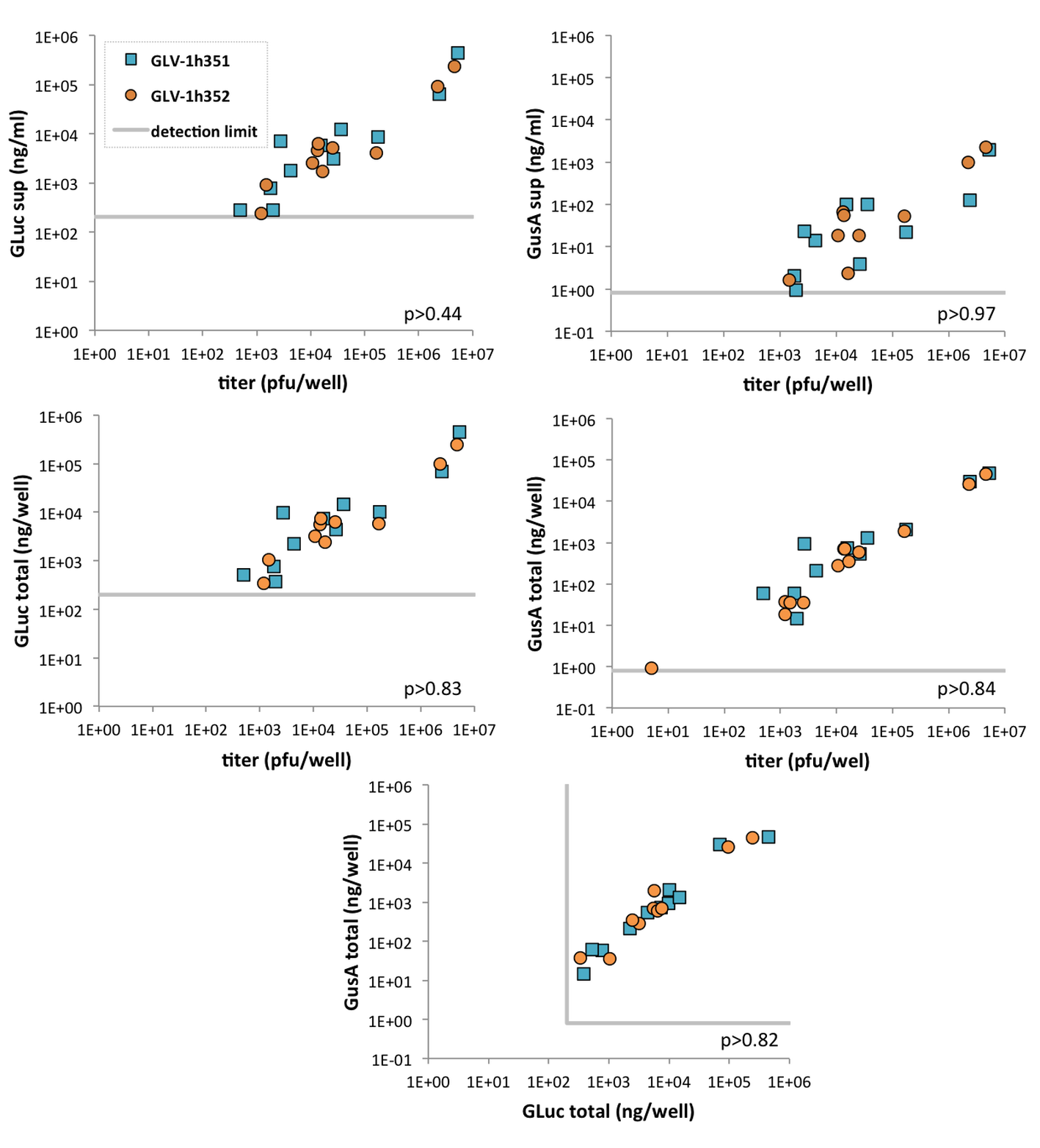

Supplement: S1 Fig — Cells (A549, CV-1 and PC-3) infected with either GLV-1h351 (blue squares) or GLV-1h352 (orange circles) produced comparable relationships of GLuc activity, GusA activity or virus titer between cells infected with GLV-1h351 or GLV-1h352. No significant differences were observed (p>0.47). (TIF) [file pone.0137573.s001.tif]

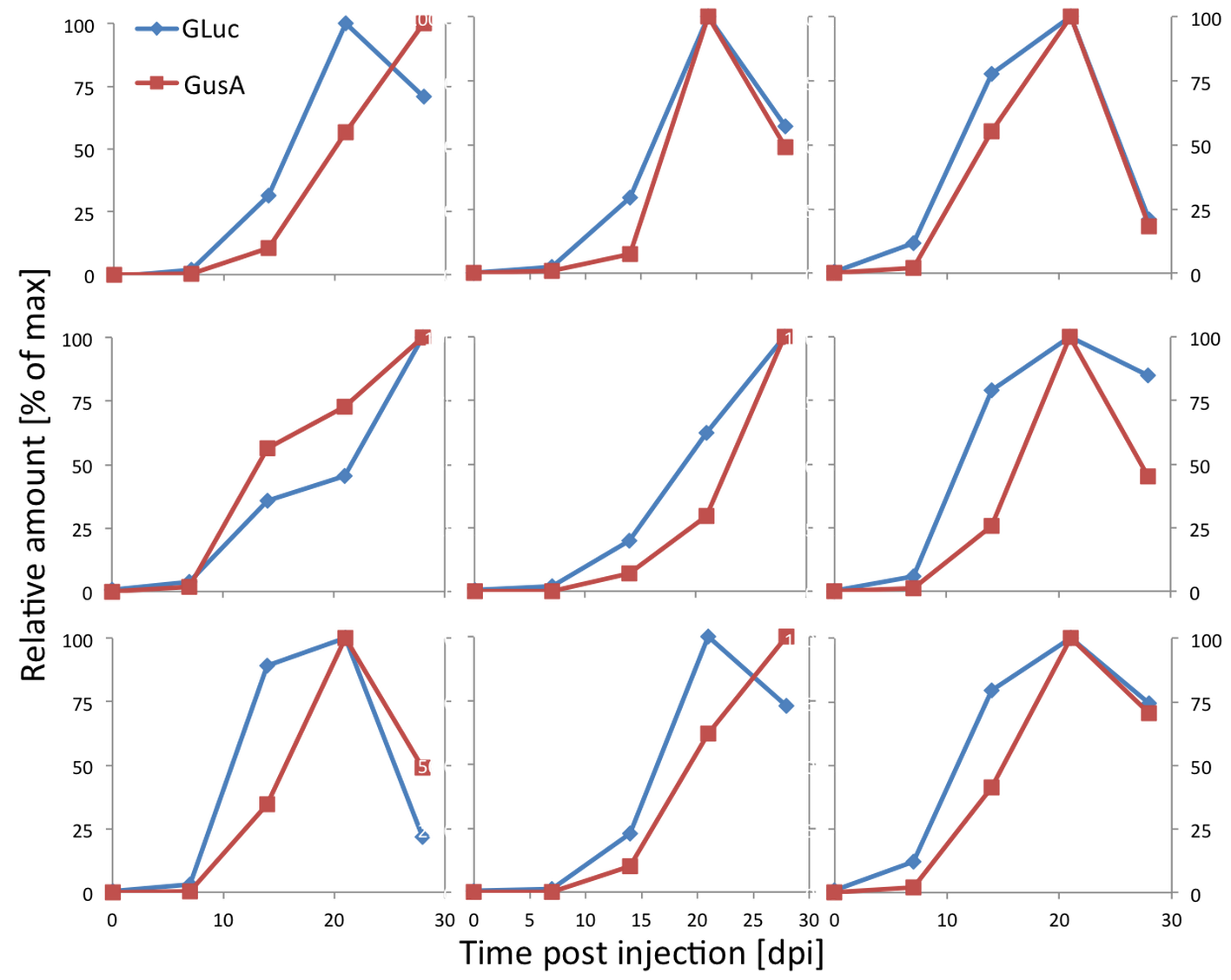

Supplement: S2 Fig — In 8 out of 9 mice, the GLuc concentration as determined by its activity increased faster than the GusA concentration (see data at 14 days post injection). (TIF) [file pone.0137573.s002.tif]
